# Optimizing antimicrobial dosing of bacterial isolates from sugar beet factories to reduce sucrose losses

**DOI:** 10.1128/aem.01646-25

**Published:** 2026-03-10

**Authors:** Gillian O. Bruni, Evan Terrell, Tia Zimmerman, Zianab Yassin, Sanjay Joshi

**Affiliations:** 1 USDA, Agricultural Research Service, Southern Regional Research Center 57578 , New Orleans, Louisiana, USA; 2 Beet Sugar Development Foundation 607026 , Fort Collins, Colorado, USA; 3 U.S. Department of Energy, Oak Ridge Institute for Science and Education 17215, Oak Ridge, Tennessee, USA; Anses, Maisons-Alfort Laboratory for Food Safety, Maisons-Alfort, France

**Keywords:** sugar beet factory, antimicrobial, MIC, sucrose savings, technoeconomic analysis

## Abstract

Bacterial growth in sugar beet factories results in sucrose losses and operational challenges during raw sugar extraction, resulting in lost revenue. Minimum inhibitory concentration (MIC) values of antimicrobial agents were measured by microdilution assay for optimized dosing to control 58 selected bacterial isolates previously obtained from sugar beet factory juices and biofilms. Hops BetaStab XL was effective against most gram-positive bacteria at 16 ppm with the exception of a few resistant *Leuconostoc* and *Weissella* outliers for which MIC values were >1,000 ppm. Sodium hypochlorite showed broad-spectrum activity against most gram-positive bacteria with MICs typically at 250 ppm, with the exception of three *Leuconostoc* sp. outliers exhibiting MIC values >1,000. Gram-negative isolates had MIC values ranging from 250 to 500 ppm. Hydritreat 2216 (peracetic acid) also exhibited broad-spectrum activity against each group, with MIC values typically lower between 31 and 250 ppm. Similarly, thyme oil and ammonium bisulfite showed similar ranges of MIC values against all isolates with MIC values ranging from 125 to >1,000 and 63 to >1,000, respectively. Elevated temperature was also examined for antimicrobial control compared to growth at 28°C. Incubation at 50°C was found to dramatically inhibit the growth of most genera tested except for *Bacillus* sp. and some *Acinetobacter* sp. Treatment with Hops BetaStab XL, sodium hypochlorite, or Hydritreat 2216 at respective MIC values during growth curve experiments inhibits growth at the earliest time points relevant for sugar beet processing in factories. Technoeconomic analysis was also conducted to estimate revenue-neutral, break-even prices for antimicrobial agent application at factory scale.

**IMPORTANCE:**

Optimal antimicrobial dosing to control key microorganisms in sugar beet factories is central to mitigating sucrose losses during sugar beet processing and preventing the additional operational challenges that often result from the production of bacterial exopolysaccharides. This study demonstrates that some antimicrobial agents effectively control key bacteria at lower concentrations while others exhibit broad variability in MIC. Additionally, elevated temperatures of factory water and juices above 50°C are another option for controlling some important microbes.

## INTRODUCTION

Sugar beet agriculture in the United States was predicted to produce 5.389 million short tons raw value (STRV) during the 2024/2025 campaign (1) and yield several billion dollars in revenue. While some sugar beets are processed fresh, more often sugar beets are stored for processing later in the campaign (2). Storage rot prior to extraction of raw sugar can be affected by harvesting, weather and storage conditions, and plant and soil microorganisms (3). During processing to extract raw sugar, microbes from infected, rotting sugar beets and associated soil are carried into the factory processing streams (4). In addition to direct sucrose losses, bacterial exopolysaccharides (EPS) often cause additional operational problems including deleterious impacts on viscosity, carbonatation, filtration, and crystallization, whereby microbial activity exerts a multi-pronged effect (5–9). The presence of dextran, a predominant bacterial EPS in sugar crop processing, has been shown to cause a decrease in the rate of sucrose crystallization (10). Dextran can also affect precipitate particle size and shape distribution during carbonatation, which may have adverse effects on juice filtration and purification (11). Levan fructans, another commonly occurring EPS in sugar crop processing (12, 13), are anticipated to have similar unwanted effects, although there is less published literature on levan fructans than dextrans.

Previous antimicrobial studies typically used sugar crop factory juices, which can vary in microbial load and composition over time and therefore may have confounded the determination of which microbes are inhibited by antimicrobial application (14, 15). More recently, research efforts have been aimed at studying individual microorganisms present in sugar beet factories in addition to whole microbial communities (4, 16–18). Together, these studies are crucial for identifying sucrose-consuming microorganisms present in factories and for obtaining isolates. In this study, antimicrobial susceptibility testing was conducted on several genera of bacterial isolates previously derived from juice and biofilm samples collected from sugar beet factories (16) to determine minimum inhibitory concentration (MIC) values to optimize antimicrobial dosing. In addition, MIC value dosing was examined for mitigation of sucrose losses by selected isolates and subsequent technoeconomic analysis. Technoeconomic analysis was used to quantify and contextualize estimated revenue losses from microbial activity at factory scale, and subsequently, to estimate reasonable costs for microbial mitigation efforts (19, 20).

## MATERIALS AND METHODS

### Microbial isolates used in this study

Bacterial isolates were isolated and identified from sugar beet factory juice and biofilm samples as previously described (16). The collection of microbial isolates from the study was named for the stakeholder organization that funded the study, Beet Sugar Development Foundation (BSDF) (16).

### Susceptibility testing of antimicrobial agents by microdilution technique

Susceptibility testing of microbial strains to antimicrobial agents was adapted from the microdilution technique (21) and performed as described previously (20). Briefly, antimicrobial agents were suspended in Tryptone sucrose yeast extract (TSY) medium (50 g/L sucrose) adapted from reference 22 and twofold serially diluted in 96-well plates in TSY medium as previously described (20). Sucrose is the predominant carbon source found in sugar beet juice (23). Sugar beets typically contain 75% water and 25% dry substance (percentages reported on an absolute basis). Of the 25% dry substance, 5% is insoluble beet marc, 18% is sucrose, and 2% is nitrogenous compounds, other sugars, minerals, and trace other components. Sugar beet diffusion juice in the factory has a dry substance content of about 15% that is predominantly sucrose (around 13% sucrose and 2% other trace components) (23).

In addition, since various antimicrobials usually differ in concentration and formulation, active ingredient parts per million (ppm) concentrations were calculated and used in the susceptibility assays. For comparison of growth at various temperatures, plate assays were set up at the same time and incubated at 28°C and 50°C for 18 h, and OD_600_ was measured in a plate reader (TECAN, Männedorf, Switzerland). MIC_50_ and MIC_90_ values were calculated for genera with 10 or more isolates tested as previously described (24). Genera with less than 10 isolates tested were listed with the range of MIC values (23).

### Technoeconomic analysis

A cost analysis is presented to provide approximations or order-of-magnitude estimates for the break-even price of antimicrobial application, based on additional revenue potentially realized by avoiding sucrose losses. The break-even price is the revenue-neutral point at which the cost of a given antimicrobial agent is taken as equivalent to the (lost) revenue associated with sugar consumption through microbial activity without antimicrobial application.

Using values for sucrose from high-pressure liquid chromatography (HPLC) data (details below) enables the computation of a slope associated with sucrose loss due to microbial activity. This, coupled with MIC determination and generalized mass flow parameters for an average beet factory, allows for the calculation of break-even price for a given antimicrobial agent on a cost per L or kg basis. Basic parameter assumptions are as follows: juice mass during processing is approximately 110% of beet mass (e.g., 1.1 tons juice per MT beet); juice and antimicrobial agent density is 1.05 kg/L; approximately 4,500 MT/day beets processed; approximately 10-min effective time for antimicrobial agent activity, based on conversations with industry personnel; and raw sugar price of 0.82 USD per kg (20, 23, 25).

### Sourcing of antimicrobial reagents

Sodium hypochlorite and thyme oil were purchased from Sigma-Aldrich (St. Louis, MO, USA). Hydritreat 2216 (22% peracetic acid) and ammonium bisulfite (ABS) were generously provided as samples from Hydrite Chemical Co. (Brookfield, WI, USA). Hops BetaStab XL (9% hops acid emulsion) was provided by Betatec (Washington DC, USA), and Magna Cide D (a proprietary carbamate formulation) was generously provided by Protech USA (Thibodaux, LA, USA). Avancid GL50 (glutaraldehyde formulation) was provided by SMC Global (New York, NY, USA).

### Growth curve analysis

Precultures of each isolate were grown in MRS medium (26) to minimize EPS production and allow for pipetting non-viscous precultures. Optical densities (OD_600_) of precultures were measured in an Eppendorf BioPhotometer (Hamburg, Germany) and normalized to 0.4 in TSY medium (50 g/L sucrose) (22). Normalized precultures were serially diluted in a 96-well plate in triplicate, so that starting OD values would be 0.05, 0.10, and 0.20 after addition of treatments. Inoculated wells were treated with either TSY medium only or TSY medium containing an antimicrobial agent at the MIC value and growth was observed over 24 h. The microbial isolates were aligned in a block design within the plates. Each block also contained triplicate wells, which were not inoculated, to be used for blanking. The outermost wells were filled only with TSY medium to mitigate evaporation of experimental wells. The plates were incubated at 28°C, 250 rpm, for 24 h in a plate reader (TECAN, Männedorf, Switzerland), which measured the OD values of wells every 10 min using Magellan Standard software, version 7.5. An average OD value was calculated for each set of triplicate values at each time point. Average OD values of blanks were subtracted from average values of conditions in each respective block.

### Growth experiments measuring sucrose consumption by HPLC

Bacterial strains were grown as precultures in MRS medium as described above and used to inoculate 50 mL TSY (50 g/L sucrose) flask cultures to 0.100 OD. Flask cultures were grown in triplicate at 28°C, 250 rpm, with 2 mL samples collected, and OD was measured at time 0 and every 30 min until the 4-h time point. Sucrose, glucose, and fructose levels were analyzed by high-pressure liquid chromatography (HPLC) as previously described (20).

## RESULTS

### Determination of minimum inhibitory concentration (MIC) values

This study was conducted to optimize antimicrobial dosing against key bacteria found in sugar beet factory juice and biofilms, several of which have been previously identified (4, 16). Therefore, relevant bacterial isolates from both juice and biofilm-derived samples representing both gram-positive and gram-negative isolates were tested for sensitivity to seven different antimicrobial agents to determine the minimum dose needed to inhibit growth, termed the minimum inhibitory concentration (MIC) value, obtained by microdilution assay in sucrose-containing medium. Tryptone sucrose yeast extract medium was used for reproducibility without any potential variation from subtle changes in beet juice composition during processing and to enable measurement of growth by OD_600_.

The results of susceptibility testing on gram-positive and gram-negative isolates are shown in Tables 1 and 2, respectively. The reported MIC values were obtained from three independent experiments in which ppm concentrations were calculated based on active ingredient concentrations. For genera with 10 or more isolates tested, MIC_50_ and MIC_90_ values were calculated, while for genera with fewer isolates, the MIC ranges are given instead (Table 3). Sodium hypochlorite and Hydritreat 2216 (peracetic acid) exhibited broad-spectrum activity against both gram-positive and gram-negative isolates. Most MIC_50_ values for sodium hypochlorite were 250 ppm. The MIC_90_ for sodium hypochlorite against *Leuconostoc* isolates was >1,000 ppm indicating phenotypic variation in sensitivity. The MIC range of other isolates was 250–500 ppm of sodium hypochlorite. Hydritreat 2216 typically inhibited microbial growth at lower doses with MIC_50_ values at 125 or 250 ppm. MIC_90_ values were 250 ppm for each applicable genus and MIC ranges for other genera were 125–250 ppm. Hops BetaStab XL was typically effective against gram-positive isolates at MIC values of 16–31 ppm with the exception of *Leuconostoc* isolates for which an MIC_90_ value of >1,000 ppm was observed indicating the presence of multiple isolates exhibiting elevated resistance to Hops BetaStab XL. Additionally, one *Bacillus* isolate, BSDF16-1, also demonstrated elevated resistance to Hops BetaStab XL with an MIC of >1,000 ppm (Table 1). Interestingly, no outliers of *Weissella* were detected with increased resistance to Hops BetaStab XL. In the case of *Leuconostoc* BSDF52-11 and BSDF25-7 isolates, both exhibited increased resistance to sodium hypochlorite and Hops BetaStab XL. The proprietary carbamate formulation MagnaCide D showed highly variable broad-spectrum activity. Both the MIC_50_ and MIC _90_ values for *Leuconostoc* were >1,000 ppm indicating general ineffectiveness of the carbamate formulation against *Leuconostoc* isolates. The MIC range for *Weissella* isolates was broad from 16 to >1,000 ppm. Magna Cide D was typically more effective against *Bacillus*, *Pantoea*, *Rahnella*, and *Acinetobacter* genera with MIC values ranging from 15 to 250 ppm (Table 3). MIC values for Avancid GL50 (glutaraldehyde) ranged from 250 to >1,000 ppm (Tables 1 and 2). Both MIC_50_ and MIC_90_ values for *Leuconostoc* isolates were >1,000 ppm. Thyme oil showed broad-spectrum activity at MIC levels often ranging from 125 to 1,000 ppm. However, MIC values were higher for several *Leuconostoc* and *Weissella* isolates between 500 and >1,000 ppm (Tables 1 and 2). The MIC_50_ and MIC_90_ values for thyme oil against *Bacillus* isolates were lower at 125 and 250 ppm, respectively (Table 3). The sensitivity of isolates to ABS was highly variable with MIC values between 63 and >1,000 ppm, in a strain-specific manner. The lowest MIC_50_ value of 125 ppm was measured for *Bacillus* isolates. Finally, no obvious trends were detected in MIC values for isolates originally obtained from juice versus biofilm.

**TABLE 1:** Antimicrobial MIC values for gram-positive bacterial juice- (white) and biofilm-derived (gray) isolates expressed as active ingredient ppm^*a*^

Microorganism	BSDF no.	NaClO^*b*^ ppm	Hydritreat 2216 ppm	Hops Betastab XL ppm	Magna Cide D ppm	Avancid GL50 ppm	Thyme oil ppm	ABS ppm
*Leuconostoc* sp.	40-1	250	63	>1,000	>1,000	>1,000	500	>1,000
*Leuconostoc* sp.	48-3	250	63	250	>1,000	>1,000	1,000	>1,000
*Leuconostoc* sp.	62-9	250	63	31	125	1,000	500	125
*Leuconostoc* sp.	47-1	250	63	125	>1,000	>1,000	250	1,000
*Leuconostoc* sp.	52-11	>1,000	250	>1,000	>1,000	>1,000	1,000	>1,000
*Leuconostoc* sp.	2-3	125	63	16	>1,000	500	500	125
*Leuconostoc* sp.	2-6	250	63	31	500	1,000	250	125
*Leuconostoc* sp.	5-1	250	125	125	125	1,000	>1,000	125
*Leuconostoc* sp.	51-4	250	250	16	31	1,000	500	500
*Leuconostoc* sp.	10-5	250	125	16	16	500	>1,000	250
*Leuconostoc* sp.	14-9	>1,000	250	250	>1,000	>1,000	1,000	250
*Leuconostoc* sp.	25-7	>1,000	250	>1,000	>1,000	>1,000	>1,000	>1,000
*Leuconostoc* sp.	31-12	250	250	31	>1,000	>1,000	500	125
*Weissella* sp.	15-1	250	125	16	>1,000	1,000	250	250
*Weissella* sp.	31-2	250	250	31	>1,000	1,000	250	1,000
*Weissella* sp.	53-1	250	250	31	>1,000	1,000	500	1,000
Weissella sp.	53-10	500	250	31	>1,000	1,000	>500	500
*Weissella* sp.	56-6	250	125	16	31	500	250	500
*Bacillus* sp.	45-1	250	125	16	31	250	125	125
*Bacillus* sp.	68-1	250	125	16	16	1,000	250	125
*Bacillus* sp.	68-7	250	125	16	63	500	125	500
*Bacillus* sp.	18-1	250	250	16	31	250	125	500
*Bacillus* sp.	18-5	250	63	16	16	250	125	63
*Bacillus* sp.	16-1	250	125	>1,000	63	250	250	500
*Bacillus* sp.	16-7	250	125	16	16	500	125	125
*Bacillus* sp.	36-6	250	63	16	16	500	250	125
*Bacillus* sp.	49-1	250	125	16	16	500	250	500
*Bacillus* sp.	49-2	250	250	16	16	500	250	250
*Peribacillus* sp.	13-5	250	125	16	31	500	125	1,000
*Peribacillus* sp.	4-7	250	250	16	31	500	125	500
*Peribacillus* sp.	4-1	250	250	16	16	500	125	500
*Peribacillus* sp.	18-2	250	250	16	31	1,000	125	500
*Peribacillus* sp.	22-11	500	250	16	31	1,000	250	500
*Peribacillus* sp.	36-1	250	250	16	16	500	125	1,000
*Peribacillus* sp.	36-9	250	250	16	16	500	250	500
*Peribacillus* sp.	51-1	250	125	16	16	500	125	500
*Peribacillus* sp.	71-1	250	250	16	16	500	250	500
*Lactococcus* sp.	3-10	250	31	16	31	500	250	1,000

^*a*^ Results were determined from the average of three independent experiments.

^*b*^ NaClO, sodium hypochlorite.

**TABLE 2:** Antimicrobial MIC values for gram-negative bacterial juice- (white) and biofilm-derived (gray) isolates expressed as active ingredient ppm^*a*^

Microorganism	BSDF no.	NaClO^*b*^ ppm	Hydritreat 2216 ppm	Hops Betastab XL ppm	Magna Cide D ppm	Avancid GL50 ppm	Thyme oil ppm	ABS ppm
*Pantoea* sp.	24-11	500	250	–^*c*^	31	>1,000	500	1,000
*Pantoea* sp.	7-5	500	250	–	16	1,000	250	1,000
*Pantoea* sp.	9-1	500	250	–	31	1,000	250	1,000
*Pantoea* sp.	45-9	500	250	–	31	1,000	250	1,000
*Pantoea* sp.	3-4	500	125	–	31	1,000	500	1,000
*Pantoea* sp.	15-4	250	250	–	31	1,000	250	500
*Pantoea* sp.	19-1	500	250	–	31	1,000	250	500
*Pantoea* sp.	19-4	250	250	–	31	1,000	250	1,000
*Pantoea* sp.	19-7	250	250	–	31	1,000	250	1,000
*Pantoea* sp.	25-2	500	250	–	125	1,000	250	1,000
*Rahnella* sp.	24-4	500	250	–	63	1,000	250	1,000
*Rahnella* sp.	26-4	500	250	–	31	500	250	1,000
*Rahnella* sp.	35-2	500	250	–	31	500	250	1,000
*Rahnella* sp.	39-3	500	250	–	63	1,000	250	1,000
*Rahnella* sp.	37-11	250	125	–	31	500	250	1,000
*Acinetobacter* sp.	21-5	250	125	–	125	500	125	1,000
*Acinetobacter* sp.	21-8	250	250	–	63	500	125	500
*Acinetobacter* sp.	38-11	500	125	–	125	500	250	1,000
*Acinetobacter* sp.	46-12	250	250	–	63	500	250	500
*Acinetobacter* sp.	60-12	250	250	–	250	500	250	1,000

^*a*^ Results were determined from the average of three independent experiments.

^*b*^ NaClO, sodium hypochlorite.

^*c*^ – indicates that the antimicrobial was not tested.

**TABLE 3:** Summary of MIC_50_ and MIC_90_ values for each genera with 10 or more strains tested^*a*^

Microorganism	Value	NaClO^*b*^	Hydritreat 2216	Hops BetaStab XL	Magna Cide D	Avancid GL50	Thyme oil	ABS
*Leuconostoc* sp.	MIC_50_	250	125	125	>1,000	>1,000	500	250
	MIC_90_	>1,000	250	>1,000	>1,000	>1,000	>1,000	>1,000
*Weissella* sp.	MIC range	250–500	125–250	16–31	16 to >1,000	500–1,000	250–500	250–1,000
*Bacillus* sp.	MIC_50_	250	125	16	16	500	125	125
	MIC_90_	250	250	16	63	500	250	500
*Peribacillus* sp.	MIC range	250	125–250	16–31	500–1,000	500–1,000	125–250	500–1,000
*Pantoea* sp.	MIC_50_	500	250	–^*c*^	31	1,000	250	1,000
	MIC_90_	500	250	–	31	1,000	500	1,000
*Rahnella* sp.	MIC range	250–500	125–250	–	31–63	500–1,000	250	1,000
*Acinetobacter* sp.	MIC range	250–500	125–250	–	63–250	500	125–250	500–1,000

^*a*^ Nine or less strains were expressed as the range of experimental MIC values. In total, 58 bacterial isolates were tested for susceptibility to seven antimicrobial agents.

^*b*^ NaClO, sodium hypochlorite.

^*c*^ – indicates that the antimicrobial was not tested.

### Growth inhibition at earlier time points relevant to sugar beet processing

**Fig 1:**
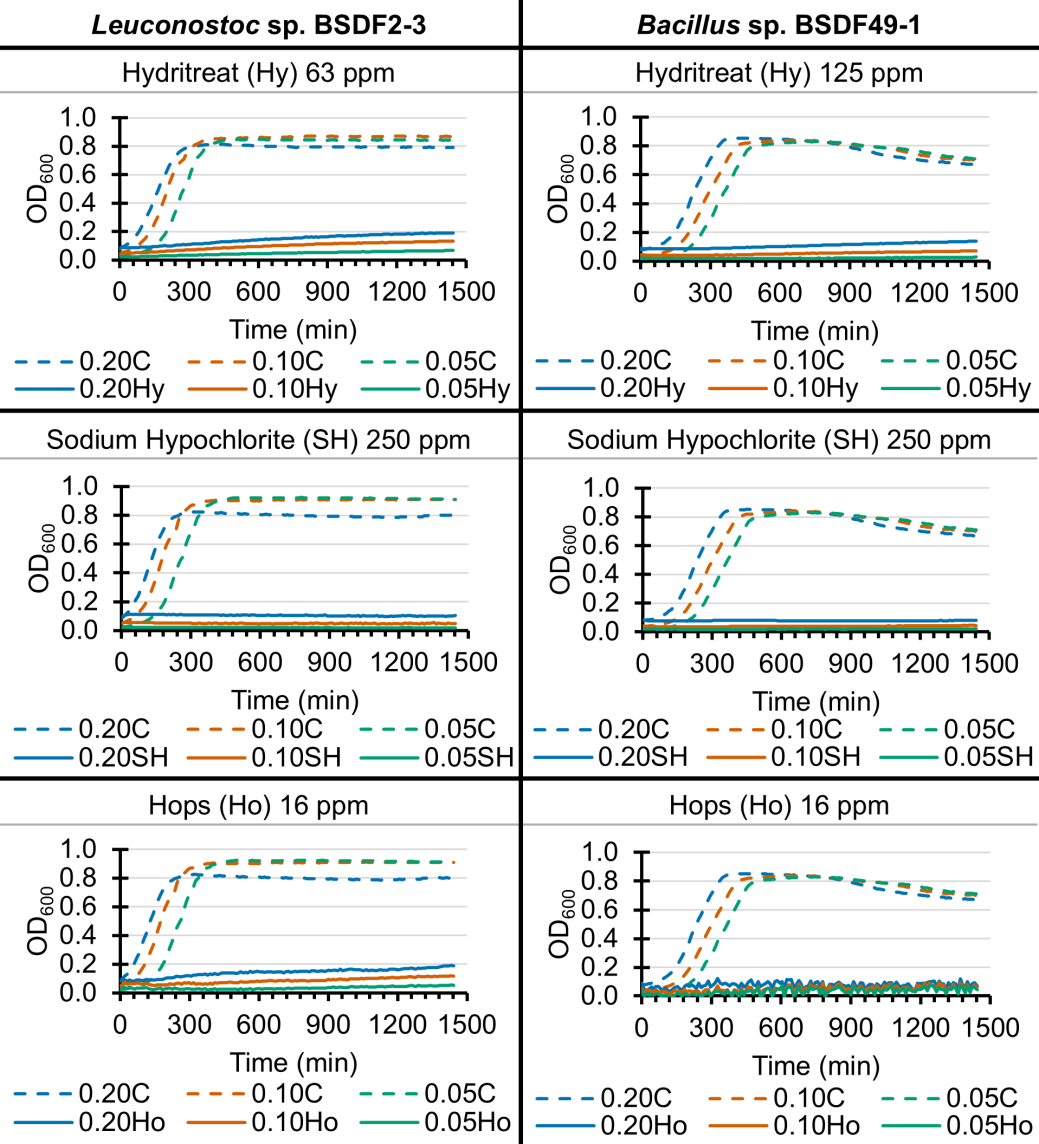
Application of antimicrobial agents at MIC inhibits growth of *Leuconostoc* sp. BSDF2-3 and *Bacillus* sp. BSDF49-1. Respective strains were inoculated at OD values of 0.05, 0.10, and 0.20 and treated with either medium (control), Hydritreat 2216, sodium hypochlorite, or Hops BetaStab XL at MIC and incubated at 28°C, 250 rpm, with OD_600_ readings every ~10 min. Growth curves of *Leuconostoc* sp. BSDF2-3 are shown on the left, and *Bacillus* sp. BSDF49-1 on the right. Antimicrobial agent and concentration used are displayed above each graph. Solid lines indicate antimicrobial agent treatment. Dashed lines indicate medium control (**C**), for comparison.

Since the microdilution assay used for susceptibility testing utilized an 18-h endpoint at which OD_600_ was measured, we sought to determine whether application of antimicrobial agents at MIC levels would inhibit growth at earlier time points more relevant to sugar beet processing in the factory, during which the time before heating to high temperatures is 10–15 min. Therefore, growth curve experiments were performed on two representative bacterial genera common to sugar beet factory juice streams (4). The *Leuconostoc* sp. BSDF2-3 and *Bacillus* sp. BSDF49-1 strains were inoculated at three starting concentrations and treated with either Hydritreat 2216, sodium hypochlorite, Hops BetaStab XL, or medium control; while the medium control resulted in an expected sigmoidal growth curve for each strain, antimicrobial treatment at the MIC value dramatically inhibited growth at the earliest observed time points (Fig. 1).

### Determination of sucrose consumption by selected bacterial isolates

**Fig 2:**
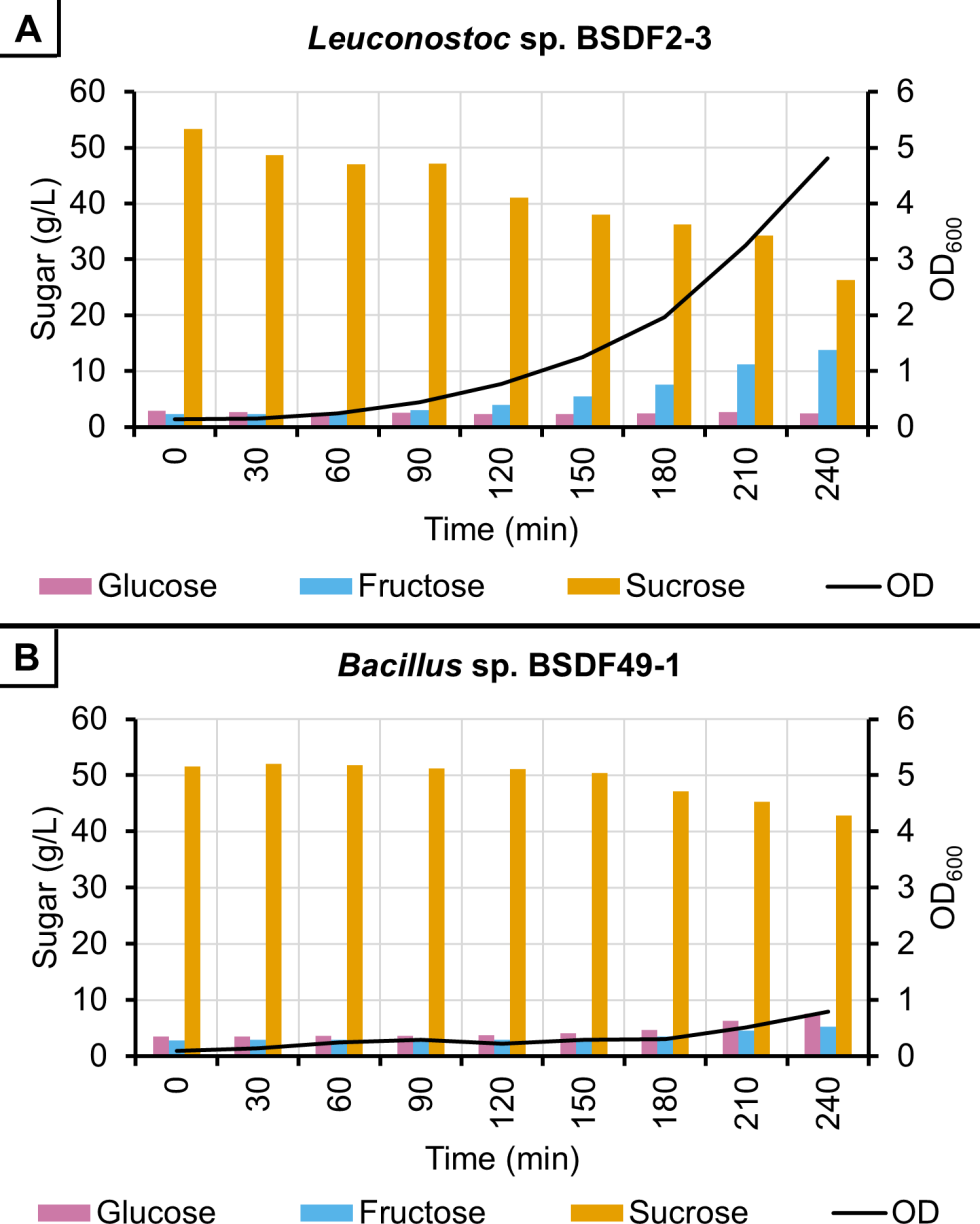
Average OD_600_ and sugar concentrations of TSY (50 g/L sucrose) medium after inoculation with (**A**) *Leuconostoc* sp. BSDF2-3 or (**B**) *Bacillus* sp. BSDF49-1. Sample size *n* = 3 for all data points, except sugar concentrations in (**B**) at 0 and 30 min where *n* = 2. All sample coefficients of variation are <12% in (**A**) and <38% in (**B**).

Next, to test the hypothesis that MIC value application of antimicrobial agents results in significant sucrose savings, early time point growth curves were conducted as flask cultures to enable sample collection for HPLC analysis of sucrose consumption (Fig. 2). *Leuconostoc* sp. BSDF2-3 and *Bacillus* sp. BSDF49-1 were used to inoculate flask cultures and sucrose consumption was measured from time 0 out to 4 h. HPLC measurements of sucrose, glucose, and fructose are shown in Fig. 2, along with associated OD_600_ values, for *Leuconostoc* sp. BSDF2-3 and *Bacillus* sp. BSDF49-1. The results in each case show an expected decrease in sucrose and corresponding increase in OD for the strains. Changes in glucose and fructose levels over time are also consistent with expected dextran (*Leuconostoc* sp. BSDF2-3) and fructan (*Bacillus* sp. BSDF49-1) producers, respectively (27).

HPLC data were used to compute a slope for sucrose consumption that equaled a rate of approximately 0.10 g/L per minute (regression *P* value ≈ 1 × 10^−6^; *R*^2^ = 0.88) by *Leuconostoc* sp. BSDF2-3. Likewise, the slope for the sucrose consumption rate of *Bacillus* sp. BSDF49-1 was measured as approximately 0.04 g/L per minute (regression *P* value ≈ 1 × 10^−3^; *R*^2^ = 0.80).

### Technoeconomic analysis of antimicrobials against microbial isolates

Using estimated sucrose losses, in conjunction with representative mass balance parameters for a sugar beet factory, enables computation of break-even cost for antimicrobial application. The break-even value is the revenue-neutral point at which the cost of hypothetical antimicrobial application is equivalent to the additional revenue realized by higher product yields in the absence of microbial sugar consumption (20). These results are given in Tables 4 and 5. From the slopes of 0.10 g/L sucrose loss per minute for *Leuconostoc* sp. BSDF2-3 and 0.04 g/L sucrose loss per minute for *Bacillus* sp. BSDF49-1, estimated break-even values are $3,900 per day for *Leuconostoc* sp. BSDF2-3 and $1,400 per day for *Bacillus* sp. BSDF49-1.

**TABLE 4:** Example calculation of an estimate for the break-even cost for control of *Leuconostoc* sp. BSDF2-3 by the NaClO antimicrobial agent^*a*^

Parameter	Value	Unit
Strain	*Leuconostoc* sp. BSDF2-3	
Antimicrobial agent	NaClO	
Estimated factory loss	1.06	kg-sucrose/MT-beet
Beet processing rate	4,500	MT-beet/day
Sugar price	0.82	USD/kg-sugar
Break-even value	3,900	USD/day
Juice and biocide density	1.05	kg/L
Juice flow rate	4,950	MT-juice/day
Antimicrobial MIC	125	ppm
Antimicrobial flow rate at MIC	589	L-biocide/day
Break-even price (vol.)	6.62	USD/L
Break-even price (mass)	6.30	USD/kg

^*a*^ This value was chosen as a representative processing rate for factories in North America, but it can be changed to account for different factory sizes. This would have the practical effect of increasing or decreasing the daily “break-even value” for larger or smaller processing rates, respectively. The beet-mass to juice-mass ratio used here is 1.0:1.1, as a representative value for factories in North America (as indicated in Materials and Methods, 1.1 MT juice per 1 MT beet). This ratio could similarly be modified as needed for different factories or beet conditions.

**TABLE 5:** Estimates for the break-even costs per liter and per kilogram for strains *Leuconostoc* sp. BSDF2-3 and *Bacillus* sp. BSDF49-1 treated with each antimicrobial agent (as per the calculation details given in Table 4)

Strain	Antimicrobial	Measured MIC	USD/L	USD/kg
*Leuconostoc* sp. BSDF2-3	NaClO (bleach)	125 ppm	6.56	6.25
*Leuconostoc* sp. BSDF2-3	Hydritreat 2216	63 ppm	13.02	12.40
*Leuconostoc* sp. BSDF2-3	Hops Betastab XL	16 ppm	51.25	48.81
*Leuconostoc* sp. BSDF2-3	MagnaCide D	1,000 ppm	0.82	0.78
*Leuconostoc* sp. BSDF2-3	Avancid GL50	500 ppm	1.64	1.56
*Leuconostoc* sp. BSDF2-3	Thyme oil	500 ppm	1.64	1.56
*Leuconostoc* sp. BSDF2-3	Ammonium bisulfite	125 ppm	6.56	6.25
*Bacillus* sp. BSDF49-1	NaClO (bleach)	250 ppm	1.19	1.13
*Bacillus* sp. BSDF49-1	Hydritreat 2216	250 ppm	1.19	1.13
*Bacillus* sp. BSDF49-1	Hops Betastab XL	16 ppm	18.56	17.68
*Bacillus* sp. BSDF49-1	MagnaCide D	16 ppm	18.56	17.68
*Bacillus* sp. BSDF49-1	Avancid GL50	500 ppm	0.59	0.57
*Bacillus* sp. BSDF49-1	Thyme oil	250 ppm	1.19	1.13
*Bacillus* sp. BSDF49-1	Ammonium bisulfite	250 ppm	1.19	1.13

The values presented for break-even cost for antimicrobials in Table 5 are given on an active ingredient basis, and therefore, do not account for the concentration of the active ingredient in a final formulated product. For example, if a computed break-even price on an active ingredient basis is $10 per L, but the antimicrobial product is 10% active ingredient, then the break-even cost for the actual product would be $1 per L (assuming negligible cost for other product ingredients). Some products have proprietary compositions that cannot be reported definitively.

In general, higher estimated break-even costs are associated with antimicrobials that have lower MIC values. Practically, this means that a factory can afford to spend more for the application of a more effective antimicrobial agent. In addition, estimated break-even costs are lower for the *Bacillus* sp. BSDF49-1 strain, due to slower sucrose consumption rate, meaning less revenue is lost to microbial activity for this strain. Practically, a consortium of microbes exists in any given factory (4, 28); the two strains chosen here were selected as overall representatives for this laboratory analysis since these two genera were in the top 10 most abundant genera previously reported (26) and these isolates were already identified and available in our microbial collection. Other genera, while of significant interest, have not been isolated by our laboratory, and many have different oxygen tolerances (anaerobes) as well as higher temperature preferences for growth such as *Thermoanaerobacter* and *Thermoanaerobacterium* (29, 30).

### Effect of elevated temperature on bacterial growth

**Fig 3:**
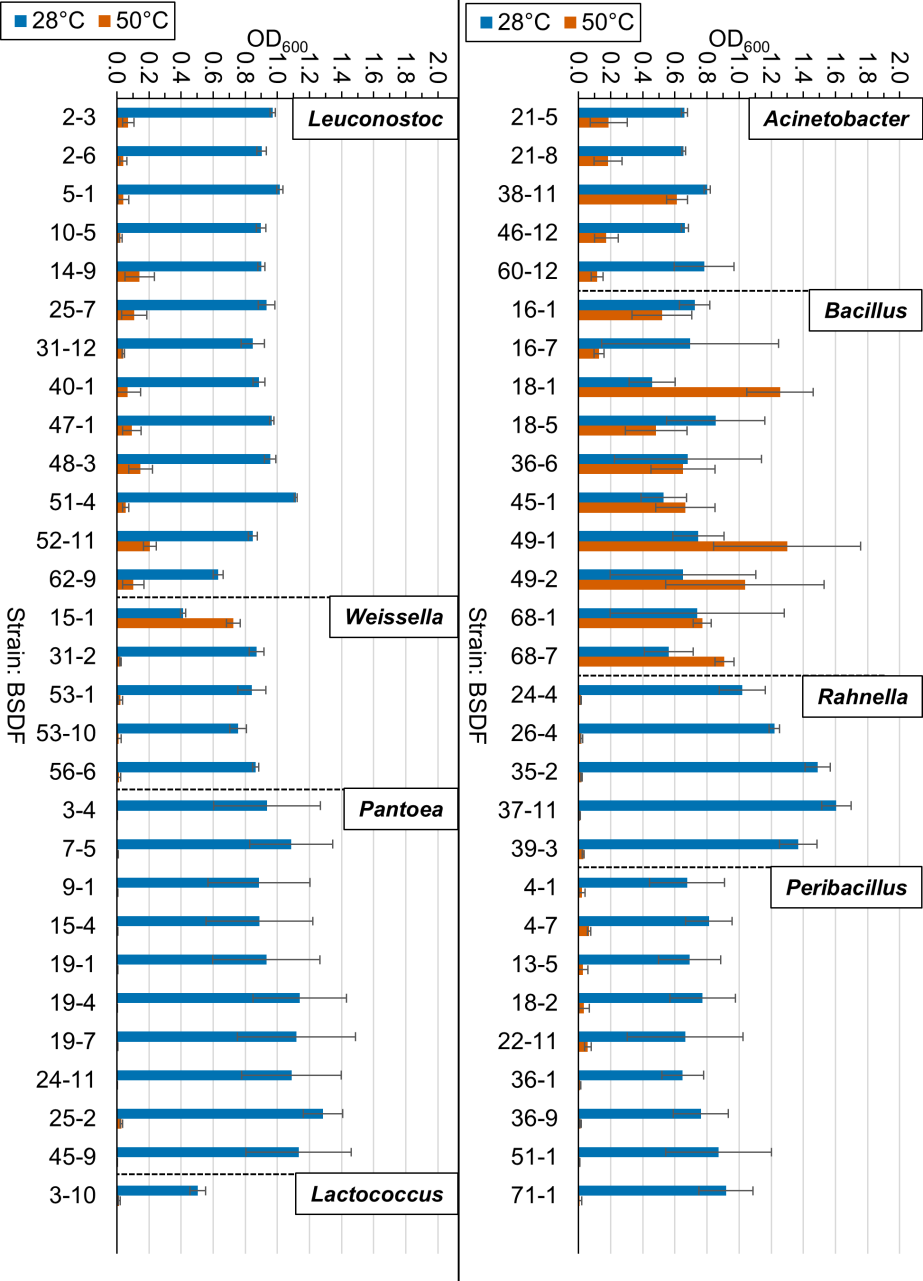
Temperature sensitivity of selected sugar beet factory isolates to elevated temperature. Growth at 28°C versus 50°C was measured by OD_600_ at 18 h. Genera shown include *Leuconostoc*, *Weissella*, *Pantoea*, *Lactococcus*, *Acinetobacter*, *Bacillus*, *Rahnella*, and *Peribacillus*.

In addition to the determination of MIC values, at the request of stakeholders, elevated temperature was evaluated for growth inhibition of several genera (Fig. 3). While practices among sugar beet factories are not always uniform, conversations with some factory operators indicate that at least in some factories, water going into the cossette mixer is preheated to 50°C. Therefore, growth assays were conducted at the standard laboratory temperature of 28°C and compared to growth at elevated temperatures such as 42°C and 50°C. Each strain exhibited normal growth at 28 °C and 42°C; for this reason, growth data at 42°C is not shown. In contrast, growth was severely inhibited in several strains at 50°C belonging to *Leuconostoc*, *Weissella*, *Peribacillus*, *Pantoea*, *Rahnella*, and *Lactococcus* genera with the exception of one *Weissella* isolate, nine *Bacillus* isolates, and one *Acinetobacter* isolate.

## DISCUSSION

This study investigated multiple antimicrobial agents commonly utilized during raw sugar beet processing in factories as well as 58 key bacterial isolates previously isolated directly from factory samples (16). Inhibition of microbial growth in factories is central to mitigating sucrose losses and preventing the additional operational challenges that often result from production of bacterial exopolysaccharides (5, 8, 31). Therefore, *in vitro* susceptibility testing was conducted to identify MIC values required for optimal dosing.

The results suggest that some commonly used antimicrobials are not likely to be effective when applied at suboptimal concentrations in factories. Moreover, many of the high MIC values measured for some bacterial-antimicrobial combinations may be cost-prohibitive and/or subject to regulatory oversight. Additionally, future studies should be aimed at elucidating molecular mechanisms of increased bacterial resistance to certain antimicrobials, as evidenced by dramatically higher MIC values. While some of these *Leuconostoc* strains demonstrate phenotypic variation in biofilm formation (32), it remains unclear whether variation in exopolysaccharide production or capacity to form biofilms may play a role in increased resistance of some factory strains, as some others have reported (33). Moreover, while several mechanisms have been reported for increased resistance of biofilms to antimicrobial control (34), the susceptibility testing conducted in the current study utilized bacterial cells grown in planktonic culture. The possible role of exopolysaccharides and biofilm formation, as well as studies integrating transcriptomic or metabolomic approaches to elucidate the molecular mechanisms of antimicrobial resistance, should be included in future research. Resistance mechanisms to various antibiotics have been reported previously involving certain *Leuconostoc* genes such as *lsaA* or formation of pentadepsipeptide with d-lactate at the C-terminal end of the muramyl pentapeptide in peptidoglycan instead of d-alanine (35–37). Further *in silico* predictions of antibiotic resistance (AR) genes in *Leuconostoc* have also been reported (38). However, these studies have not been specifically conducted on the factory-derived strains used in our study since these were only recently isolated and reported and are still relatively understudied (16, 39).

Notably, it may also be more practical for factories to use antimicrobials that work effectively at lower concentrations. Moreover, it may not be cost-effective to apply antimicrobial agents at sub-optimal doses that do not inhibit the growth of key microbes. While this study utilized individual bacterial isolates for MIC measurements, future work is needed to determine the typical microbial load present in sugar crop factory streams, its variation, and how this may affect the overall susceptibility of microbial communities (including yeast and fungi) to antimicrobial control strategies. Further, factory studies are needed to test the efficacy of MIC level dosing on juices instead of individual microbial cultures.

Notably, while this study addressed antimicrobial dosing against bacterial isolates from sugar beet factories, the impact of other microbes such as yeast should not be overlooked since yeasts are voracious sucrose consumers in sugar beets storage piles and processing streams (3, 20). Potential impact of microbial interactions and sucrose consumption by additional microorganisms during sugar beet processing as well as sucrose savings realized by improved antimicrobial strategies may also be underestimated. Future studies are needed to assess the effect of additional microbial genera and elevated temperature on growth and sucrose consumption rates by less characterized microbes further downstream in factory processing streams.

Additionally, the results showing temperature sensitivity of several isolates from key genera such as *Leuconostoc*, *Weissella*, *Pantoea*, *Rahnella*, and *Peribacillus* suggest that heating water and sugar beet factory juices at the earliest points in processing to 50°C would be advantageous in controlling several of these sucrose-consuming microbes. However, the results are also cautionary, showing thermotolerance by *Bacillus* isolates especially. This may explain the relative abundance of *Bacillus* reported by others in sugar beet factory juices (4). Additionally, the presence of other thermophiles such as *Geobacillus*, *Thermoanaerobacterium*, and *Thermoanaerobacter* indicated by microbiome analysis of diffuser juice has been reported (4). Therefore, an optimal antimicrobial strategy may incorporate elevated temperature as well as antimicrobial agents that successfully target thermotolerant microbes such as *Bacillus* as well as thermophiles. Future studies are also needed to obtain thermophilic bacteria from sugar beet factories to determine optimal antimicrobial strategies. However, the addition of peracetic acid or hops acid during the initial diffusion stage in combination with elevated temperature would likely be advantageous. Additionally, pulp-press water that is returned to the diffuser can be heated to high temperature (approximately 70–90°C) for a length of time to introduce microbial control in this flow stream, prior to re-mixing in the lower-temperature (approximately 50–65°C) diffuser (23). Furthermore, unit operations within a given factory that are downstream of diffusion (e.g., liming and purification, sedimentation and filtration, evaporation, and crystallization) are generally operated at high temperature with increasing sugar concentrations, where microbial control and elevated viscosity is less of a concern (23).

TEA is central to the determination of which antimicrobial is most cost-effective. Reframing MIC metrics in economic terms at factory scale allows for an alternative and practical way to compare among different potential antimicrobials. This also enables factory personnel to consider the cost of antimicrobial application relative to the costs associated with additional heating of processing streams for enhanced microbial control. It is difficult to exactly compute a cost associated with process heat directly in this study, due to the complicated nature of heat integration and transfer in countercurrent flow diffusion systems (40), within the context of a whole sugar beet factory. However, any given factory may be able to perform these engineering calculations as needed to perform cost comparisons for different modes of microbial control. Further, it is worth noting that antimicrobial application at a revenue-neutral, break-even cost based on sucrose savings is still likely advantageous due to indirect benefits related to microbial control. These were discussed previously, regarding the effects of bacterial EPS on parameters like viscosity, filtration, and crystallization (5–8). Furthermore, the quantitative approaches presented in this work to systematically study bacterial isolates and antimicrobial agents support the sugar beet industry by providing a framework for data-based decision-making for factory engineering and operations. Coupling applied microbiology work with factory-scale mass balance parameters facilitates estimations related to economic feasibility, which supports improved efficiency and profitability.

Finally, a broad range of microbes exists in the factory juice microbiome more than previously recognized (4, 16). Careful study using individual microbial isolates enables focused, reproducible measurements of susceptibility profiles to antimicrobial agents for optimized microbial control to reduce sucrose losses. This study demonstrated that elevated temperature applied early during sugar beet processing would be advantageous to inhibit growth of microbes including *Leuconostoc*, *Weissella*, *Pantoea*, *Rahnella*, and *Peribacillus,* while antimicrobial treatment such as hops acid or peracetic acid should be applied to inhibit thermotolerant bacteria such as *Bacillus* that are likely to persist further throughout processing. Additionally, peracetic acid demonstrated effective broad-spectrum antimicrobial activity against gram-positive and gram-negative bacteria at lower MIC values relative to other antimicrobial agents. Furthermore, the peracetic acid product tested in this study is generally recognized as safe (GRAS) for application to components of human food. This approach may also have broader implications for other food-related industries combating microbial issues related to product quality (41, 42).
